# Environmental Radiofrequency Electromagnetic Fields Exposure at Home, Mobile and Cordless Phone Use, and Sleep Problems in 7-Year-Old Children

**DOI:** 10.1371/journal.pone.0139869

**Published:** 2015-10-28

**Authors:** Anke Huss, Manon van Eijsden, Monica Guxens, Johan Beekhuizen, Rob van Strien, Hans Kromhout, Tania Vrijkotte, Roel Vermeulen

**Affiliations:** 1 Institute for Risk Assessment Sciences, Utrecht University, Utrecht, The Netherlands; 2 Institute for Social and Preventive Medicine, Bern, Switzerland; 3 Department of Epidemiology, Documentation and Health Promotion, Public Health Service of Amsterdam (GGD), Amsterdam, The Netherlands; 4 Center for Research in Environmental Epidemiology, Barcelona, Spain; 5 Department of Environmental Health, Public Health Service of Amsterdam (GGD), Amsterdam, The Netherlands; 6 Department of Public Health, Academic Medical Center, University of Amsterdam, Amsterdam, the Netherlands; 7 Julius Centre for Public Health Sciences and Primary Care, University Medical Centre, Utrecht, The Netherlands; Institute for Health & the Environment, UNITED STATES

## Abstract

**Background:**

We evaluated if exposure to RF-EMF was associated with reported quality of sleep in 2,361 children, aged 7 years.

**Methods:**

This study was embedded in the Amsterdam Born Children and their Development (ABCD) birth cohort study. When children were about five years old, school and residential exposure to RF-EMF from base stations was assessed with a geospatial model (NISMap) and from indoor sources (cordless phone/WiFi) using parental self-reports. Parents also reported their children’s use of mobile or cordless phones. When children were seven years old, we evaluated sleep quality as measured with the Child Sleep Habits Questionnaire (CSHQ) filled in by parents. Of eight CSHQ subscales, we evaluated sleep onset delay, sleep duration, night wakenings, parasomnias and daytime sleepiness with logistic or negative binomial regression models, adjusting for child’s age and sex and indicators of socio-economic position of the parents. We evaluated the remaining three subscales (bedtime resistance, sleep anxiety, sleep disordered breathing) as unrelated outcomes (negative control) because these were *a priori* hypothesised not to be associated with RF-EMF.

**Results:**

Sleep onset delay, night wakenings, parasomnias and daytime sleepiness were not associated with residential exposure to RF-EMF from base stations. Sleep duration scores were associated with RF-EMF levels from base stations. Higher use mobile phones was associated with less favourable sleep duration, night wakenings and parasomnias, and also with bedtime resistance. Cordless phone use was not related to any of the sleeping scores.

**Conclusion:**

Given the different results across the evaluated RF-EMF exposure sources and the observed association between mobile phone use and the negative control sleep scale, our study does not support the hypothesis that it is the exposure to RF-EMF that is detrimental to sleep quality in 7-year old children, but potentially other factors that are related to mobile phone usage.

## Introduction

Sleep difficulties in children are common; 20%-40% of children are reported to experience such problems [1, 2]. Sleep difficulties in children are a major cause of morbidity and have been associated with poor school performance [3], behaviour problems [4], neurocognitive effects [5] as well as obesity [6].

Radiofrequency electromagnetic fields (RF-EMF) are emitted by a range of modern telecommunication devices, such as mobile and cordless phones and their base stations, transmitters or wireless internet connections [7]. Persons who attribute symptoms to exposure to electromagnetic fields frequently report experiencing sleep problems [8–10]. Affected persons usually list a variety of suspected sources, of which mobile phone base stations and the own use of mobile phones are very frequently named [9, 11, 12]. Previous studies evaluating potential effects from RF-EMF on sleep have been inconsistent: experimental studies have reported effects on the recording of brain electrical activity as measured with the electroencephalography (EEG) [13], but effects on macrostructure of sleep (e.g. duration of sleep stages) was inconsistently related to RF-EMF exposure [14–16].

A few observational studies have been performed that assessed the effect of environmental RF-EMF exposure on self-reported sleep quality: In adults, neither measured nor modelled environmental RF-EMF exposure was reported to be associated with sleep disorders [17–20]. Regarding mobile phone use, one cross-sectional study reported an increased risk of sleeping problems in 8–12 year old children who used a mobile phone at least daily [21]. This is in line with the scientific literature which overall suggests a negative effect from the use of electronic media, including mobile phones, on sleep in children [22]. Several mechanisms (e.g. arousal, blue light exposure) underlying this effect have been suggested, but also the exposure to RF-EMF [22]. Exposure to RF-EMF occurs through various sources, such as from base stations, wireless internet connections (WiFi) or mobile phone use. Aim of our prospective study was to evaluate if exposure to environmental RF-EMF from outdoor or indoor sources and the use of mobile or cordless phones was associated with parental reported quality of sleep of 2,361 children, aged 7 years.

## Methods

Our analysis was based on data from the Amsterdam Born Children and their Development (ABCD) cohort (www.abcd-study.nl), a community-based prospective cohort study that examines the relationship of maternal lifestyle and psychosocial determinants during pregnancy to multiple aspects of development and health of the child. The ABCD Study has been described in detail elsewhere [23]. Briefly, between January 2003 and March 2004, 8,266 pregnant women were enrolled during their first prenatal visit to an obstetric care provider and filled out the pregnancy questionnaire. When children were 5 years old, parents of 4488 children filled in a questionnaire providing information on potential confounders and additionally, children participated in cognitive testing. In 2011, when children were about 7 years old, 3,460 parents filled in a questionnaire that inquired about sleep difficulties and exposure-relevant questions. Exposure relevant questions related to age 5, as this was the time that the children participated in a cognitive testing survey and we were interested to study the association between exposure to EMF-RF and cognitive functioning. Information for our analysis was available for 2,361 children (29% of the original cohort) who had participated both at age 5 and age 7. Approval of the study was obtained from the Central Committee on Research involving Human Subjects in the Netherlands, the Medical Ethical Committees of the participating hospitals, and from the Registration Committee of the Municipality of Amsterdam. Participating mothers gave written informed consent, and in addition, all data were analysed in an anonymised way.

### Environmental radiofrequency electromagnetic field exposure

Environmental RF-EMF exposure from mobile phone base stations at children’s homes was estimated using the 3D geospatial radio wave propagation model NISMap for the address the child lived at the age of 5 [24, 25]. NISMap computes the field strengths of different frequency bands. The model uses detailed information of mobile phone base station antennas (containing the location, frequency, direction, power level, height, vertical tilt and pattern of each antenna) and the 3D geometry of the urban environment to compute the RF-EMF for any location in 3D-space. The 3D geometry consists of a box model of all buildings in the Netherlands, retrieved by combining building data of the Netherlands’ Mapping Agency (Kadaster) and the Netherlands’ elevation model (Actueel Hoogtebestand Nederland 2, AHN2). NISMap uses the box model to estimate shielding and diffraction by buildings. Since we were primarily interested in mobile telephony-associated stationary sources, we assessed the downlink component of the three mobile phone communication bands (GSM900, GSM1800 and UMTS), using a countrywide mobile phone base station data set from 2011. Home addresses of the children were geocoded using Dutch cadastral data. As RF-EMF differs with height, we estimated height above ground of the room in which the children spend the majority of their time, i.e. the bedroom. We therefore collected information on the floor level of the bedroom and the total number of floors using parental questionnaires, and obtained the height of the building from our 3D-building data. Next, we computed the height above ground with the following formula: Height = (BuildingHeight / TotalNrFloors) * BedroomFloor + 1.5. For this location, the downlink exposure of the mobile phone bands was estimated. The model has been validated with outside as well as inside measurements and has been shown to predict the relative ranking of the downlink exposure reasonably well [24, 25]. Environmental RF-EMF levels were categorized at the 50^th^ and 90^th^ percentile of the modelled exposure into low, medium and high exposed, respectively. Please note that at the time of the study (2011), Long Term Evolution technology (LTE, also called 4G) was not yet implemented in the Netherlands.

There is no known mechanism of how RF-EMF exposure could cause sleeping problems. Thus, it is also unclear if total exposure received during a day, or home (night) exposure would be more relevant.For a subset of 511 children, we additionally performed measurements of RF-EMF fields in the classroom where they were placed at age 5. These measurements were performed in a subgroup of children living in Amsterdam; the measurements have been previously described elsewhere [25]. In these schools, we always performed measurements in two classrooms, but assessed the exact location of further classrooms of ABCD children if they were placed in this particular school. Therefore, for another subset of 371 children we did not have measurements, but used the exact location information on where the classrooms were located within the building to model exposure in the classrooms. We used exposure at school and at home to calculate average exposure received. For all other children we didn’t have the exact classroom location and were thus unable to evaluate their daytime exposure.

Regarding indoor sources, questions on ownership of a cordless phone and WiFi at home (“Did you have a cordless phone at home?” and “Did you have WiFi at home (wireless internet)?”, respectively) were asked to the parents and referred to the time the child was five years old.

### Child mobile phone use

Regarding mobile and cordless phone use at the age of 5, we asked “On average, how many calls did your child do with a mobile phone (this relates to all mobile phones, not only his/her own mobile phone)?” and “On average, how many calls did your child do with a cordless phone?”, respectively. Answer categories were: none, <1call/week, 1call/week or more (specifying the number of calls), and not known. We categorised the frequency of both phone uses into 4 categories (none, <1call/week, 1-2calls/week, ≥3calls/week). Some mothers answered their child had 1 call/week or more but did not specify the number of calls (n = 26 (1%) for mobile phone and n = 48 (2%) for cordless phone).

We imputed these number of calls in order to classify these individuals into 1–2 calls/week or ≥3 calls/week categories; predictor variables for the imputation were parental countries of birth, maternal age, maternal educational level, parental financial situation and maternal occupational attainment.

We also evaluated the child’s current ownership of a mobile phone at age 7, which was also asked to the parents (“Does your child currently own a mobile phone?”; answer categories were yes or no).

### Sleep problems

When children were 7 years old, parents completed the Child Sleep Habits Questionnaire (CSHQ), a 33-item questionnaire developed as a sleep screening tool for school-aged children.[2] Parents were asked to report their child’s average sleep behaviour during the last typical week. The CSHQ allows for 8 subscales or domains: sleep-onset delay (1 item), sleep duration (3 items), night wakening (3 items), parasomnias (7 items), daytime sleepiness (8 items), bedtime resistance (6 items), sleep anxiety (4 items), and sleep disordered breathing (3 items); two of the items on the bedtime resistance and sleep anxiety subscales are identical [26]. Items were rated on a 3-point scale; usually (5 to 7 times per week), sometimes (2 to 4 times per week), and rarely (0 to 1 time per week). A higher score indicates more sleep problems. Good reliability and validity have been reported for the Dutch translation of the CSHQ [26]. We additionally inquired about usual bedtimes and wakeup times of the children and used this information to calculate total time in bed in hours per night.

### Covariates

At enrolment, information on maternal age and parental countries of birth was obtained by a questionnaire completed by the mother. A postal questionnaire when the children were 5 years old provided information regarding maternal educational level (high (university degree), medium (higher secondary school), and low (lower secondary school)), the parents’ financial situation (“a lot to spare; a little to spare; just enough; to use or dip into the savings, combined with: to go into the red or into debt”), paternal age and number of brother/sisters of the index child.

### Statistical analysis

Maternal and child characteristics according to the categories of environmental RF-EMF exposure from mobile phone base stations, indoor sources and mobile or cordless phone use at age 5 were described using arithmetic means or percentages. All applied tests were either chi-square, Kruskal-Wallis or non-parametric tests for trend.

For this study, we hypothesised that if exposure to RF-EMF had an effect on sleep, it would affect those subscales that evaluated problems falling asleep or sound sleep as such. Hence, we selected the following five sleep problems subscales *a priori* as potentially related to RF-EMF exposure: sleep-onset delay, night wakening, parasomnias, daytime sleepiness, and sleep duration. We also *a priori* hypothesised that exposure to RF-EMF would be unlikely to cause the behavioural or medical-condition related sleep problems bedtime resistance, sleep anxiety or sleep disordered breathing. These remaining three subscales were therefore explored as negative controls, to assess if potentially unmeasured confounding might have affected our analysis. We also evaluated the total sleep difficulties score.

Since the sleep-onset delay subscale only includes one item, we categorized this subscale in usually or sometimes vs. rarely. We performed logistic regression models to examine the association between categories of RF-EMF exposure and the sleep onset delay subscale. The other sleep problems subscales were count variables and a negative binomial distribution fitted better with these subscales. We therefore performed binomial negative regression models to examine the association between categories of RF-EMF exposure and the sleep subscales. In these models, accounting for the respective other type of RF-EMF exposure (adjusting base station exposure for indoor exposure, or mobile phone use for cordless phone use, and vice versa) did not materially affect results, and shown are results where the other exposures were not added to the model. We performed linear regression models to examine the association between categories of RF-EMF exposure and total sleep time in hours per night.

We adjusted all models for potential confounder variables including age and sex of the child, mothers and fathers country of birth, maternal and paternal age, maternal educational level, parental financial situation, and the child’s number of siblings. We additionally adjusted the association between mobile phone use and sleep onset delay, sleep duration, night wakenings and parasomnias for other factors that have been described to affect sleep in children and that could potentially be related to mobile phone use: Whether the child lived with both of its parents or not [27], amount of TV watching [28], playing computer games [29] and playing outside [30], parental reported behaviour problems of the child (Strength and Difficulties Questionnaire, SDQ total score) [31], or doctor-diagnosed asthma [27]. Information on these factors was available for age five, with the exception of asthma, which was only available at age seven.

We also performed a sensitivity analysis on the evaluated sleep scores. We repeated our analysis on categories of environmental RF-EMF exposure at home for the subset of children where we additionally had measurements or modelled exposure in classrooms: For these children, we combined the mobile phone base station exposure at these two places into a single measure of total exposure to RF-EMF. We used the best available measure of exposure at school that we had for the children, meaning that we used measured values where we had measurements and modelled exposure for the remaining subgroup. To combine the exposure, we first accounted for the slight overestimation of our modelled compared to the measured exposure (factor 1.29) [25]. We then combined home exposure by weighing it 6/7^th^ and school exposure by 1/7^th^. This corresponds to the roughly 24hrs that children of that age spend at school per week in the Netherlands. We then repeated the fully-adjusted analysis for our target sleep subscales as for home exposure described above.

All analyses were performed using Stata version 12 (StataCorp, College Station, Texas, USA).

## Results

Of the participants, for 116 children (5%) we were not able to geocode the home address and could therefore also not model the exposure to environmental RF-EMF from base stations at home. 50 parents (2%) did not provide information on presence or absence of indoor sources, 60 parents (3%) did not report on their child’s mobile phone use and 348 (15%) did not report on cordless phone use.

Children were on average 7.4 years old, with a range of 6.7–8.5 years; 49% were girls. By definition, 10% of the group fell into the highest category of exposure from mobile phone base stations, 40% into the medium exposed category and 50% into the lowest exposed category. 68% of the parents had both a cordless phone and a wireless internet connection at home, only 5% had neither of these indoor sources. At least some degree of mobile phone use was reported for 47% of the children, for cordless phones this percentage was 72%. Families that had both indoor sources WiFi and cordless phones were more likely to be of higher socio-economic position compared to families that had neither. Mobile phone ownership, and mobile and cordless phone usage of the child was higher in families of lower socio-economic position. Characteristics of the study population across exposure categories are given in Tables 1 and 2. Regarding the duration of mobile phone calls in minutes, parents reported generally short durations: usually 1,2 or 5 minutes with a median of 2 minutes (interquartile range (IQR) 2–5). For cordless phone use median duration was 3 minutes (IQR 2–5). There was too little contrast in duration of mobile phone calls for a meaningful analysis.

**Table 1:** Maternal, paternal, and child characteristics by RF-EMF^a^.

	RF-EMF exposure from base stations, age 5				RF-EMF exposure from WiFi/cordless phones, age 5					mobile phone owernership at age 7		
	Low	Medium	High	*P*-trend	both	WiFi no, CP yes	WiFi yes, CP no	neither	*P*-diff	no	yes	*P*-diff
**Child characteristics**	** **	** **	** **		** **	** **	** **	** **				
N	1094	919	237		1607	438	158	108		2223	108	
Age (years)	7.4	7.4	7.4	0.68	7.4	7.4	7.3	7.5	0.03	7.4	7.6	<0.001
Sex (% girls)	50	47	52	0.94	50	46	46	44	0.24	48.6	49.1	0.928
**Maternal characteristics**												
Age (years)	39.7	39.8	39.6	0.55	40	39.7	38.6	37.5	<0.001	39.8	38.6	0.006
Country of birth (% non Dutch)	17	23	19	0.01	16	25	20	44		19	36.1	<0.001
Educational level (%) high	74	69	77		78	62	65	46		73.5	41.7	
medium	19	20	13		17	25	22	27		18.9	27.8	
low	7	11	9	0.35	6	13	13	28	<0.001	7.7	30.6	<0.001
**Paternal characteristics**												
Age (years)	42.2	42.6	42.7	0.13	42.3	43.3	41.1	41.9	0.000	42.4	42.2	0.775
Country of birth (% non Dutch)	21	25	24	0.10	18	32	31	51	<0.001	21.8	49.5	<0.001
**Parental financial situation**												
A lot to spare	27	26	30		31	18	22	14		27.5	12	
A little to spare	40	39	37		41	38	40	26		40.1	36.1	
Just enough	22	23	20		18	30	26	43		21.7	29.6	
To use or dip into the savings	5	5	7		6	5	3	6		4.9	8.3	
To go into the red or into debt	6	7	6	0.64	5	9	10	12	<0.001	5.8	13.9	<0.001

^a^Values are percentages for the categorical variables and arithmetic means for the continuous variables

CP: Cordless phone; P-diff = P-value for group differences based on chi-square or Kruskal-Wallis tests; P-trend = P-value for non-parametric test for trend

**Table 2:** Maternal, paternal, and child characteristics by mobile and cordless phone use.

	Mobile phone use at age 5^a^					Cordless phone use at age 5^a^				
	none	less than once per week	1–2 per week	3 times or more per week	*P*-trend	none	less than once per week	1–2 per week	3 times or more per week	*P*-trend
**Child characteristics**										
N	1196	863	140	102		314	1154	343	202	
Age (years)	7.4	7.4	7.4	7.4	0.40	7.4	7.4	7.4	7.4	0.34
Sex (% girls)	45	53	51	55	0.002	39	50	52	59	<0.001
**Maternal characteristics**										
Age (years)	40.1	39.5	39.0	38.8	<0.001	40.0	40.0	39.8	39.5	0.39
Country of birth (% non Dutch)	21	17	19	25	0.50	21	17	13	27	0.44
Educational level (%) high	74	73	68	55		71	78	74	59	
medium	18	20	21	32		19	17	19	28	
low	9	7	11	14	0.001	10	5	7	12	0.002
**Paternal characteristics**										
Age (years)	42.8	42.2	40.9	41.0	<0.001	43.0	42.5	42.1	42.4	0.41
Country of birth (% non Dutch)	22	21	29	43	<0.001	21	18	20	33	0.001
**Parental financial situation**										
A lot to spare	28	27	24	22		27	28	34	23	
A little to spare	42	38	40	35		40	43	37	36	
Just enough	21	23	23	24		23	19	20	25	
To use or dip into the savings	4	7	2	10		5	6	4	7	
To go into the red or into debt	6	5	10	9	0.003	6	5	7	9	0.27

^a^Values are percentages for the categorical variables and arithmetic means for the continuous variables

P-trend = P-value for trend

Overall sleep scores are given in Table 3. On average, children went to bed at around 8pm and woke up shortly before 7am.

**Table 3:** Sleep scores.

CSHQ score	N	mean	SD
**Bedtime resistance**	2279	7.0	1.7
**Sleep onset delay**	2336	1.4	0.7
**Sleep duration**	2290	3.6	1.0
**Sleep anxiety**	2268	4.8	1.3
**Night wakening**	2246	3.5	1.0
**Parasomnias**	2246	8.5	1.6
**Sleep disordered breathing**	2297	3.3	0.7
**Daytime sleepiness**	2174	11.5	2.9
**Total score**	1956	41.2	5.6

Results of the analysis of RF-EMF on sleep adjusted for potential confounders are shown in Table 4, and sex and age-adjusted results are presented in S1 Table. Two of our five *a priori* selected sleep scores, were associated with exposure to estimated environmental RF-EMF from mobile phone base stations: Children exposed to higher levels of base station exposure were more likely to have a higher, less favourable sleep duration score, and a lower, more favourable parasomnia score. Exposure to indoor sources was not associated with sleep scores.

**Table 4:** Fully-adjusted association between RF-EMF exposure sources and those sleeping problems *a priori* hypothesized to be potentially related to RF-EMF exposure.

	Sleeping problems									
	Sleep onset delay		Sleep duration		Night wakenings		Parasomnias		Daytime sleepiness	
	OR	(95% CI)	IRR	(95% CI)	IRR	(95% CI)	IRR	(95% CI)	IRR	(95% CI)
**Environmental RF-EMF exposure from mobile phone base stations at home**										
<50th perc.	1.00		1.00		1.00		1.00		1.00	
50-90th perc.	1.05	(0.77 to 1.42)	1.12	(0.96 to 1.30)	1.03	(0.86 to 1.23)	1.04	(0.94 to 1.14)	1.02	(0.94 to 1.11)
>90th perc.	0.90	(0.55 to 1.49)	1.22	(0.97 to 1.54)	0.87	(0.65 to 1.17)	0.82	(0.69 to 0.97)	0.95	(0.83 to 1.09)
p-val trend		0.89		0.05		0.59		0.19		0.75
**RF-EMF indoor sources (Cordless phone/Wi-Fi)**										
None	1.21	(0.57 to 2.56)	1.32	(0.94 to 1.86)	0.98	(0.64 to 1.50)	1.02	(0.80 to 1.30)	1.00	(0.82 to 1.22)
WiFi yes, cordless phone no	1.04	(0.71 to 1.52)	0.92	(0.76 to 1.11)	1.03	(0.83 to 1.28)	0.93	(0.82 to 1.05)	0.93	(0.84 to 1.04)
WiFi no, cordless phone yes	1.22	(0.70 to 2.14)	1.14	(0.86 to 1.52)	0.71	(0.49 to 1.04)	0.91	(0.75 to 1.11)	0.95	(0.81 to 1.11)
Both	1.00		1.00		1.00		1.00		1.00	
**Mobile phone use at age 5**										
No use	1.00		1.00		1.00		1.00		1.00	
less than once per week	0.94	(0.69 to 1.28)	1.26	(1.08 to 1.46)	1.11	(0.93 to 1.33)	1.11	(1.01 to 1.23)	1.04	(0.96 to 1.13)
1–2 per week	1.42	(0.81 to 2.49)	1.42	(1.07 to 1.88)	0.77	(0.53 to 1.12)	0.93	(0.76 to 1.14)	1.14	(0.97 to 1.34)
3 times or more per week	1.86	(0.97 to 3.55)	1.43	(1.00 to 2.03)	1.51	(1.02 to 2.23)	1.30	(1.04 to 1.63)	1.10	(0.91 to 1.34)
p-val trend		0.12		0.001		0.21		0.05		0.08
**Cordless phone use at age 5**										
No use	1.00		1.00		1.00		1.00		1.00	
less than once per week	0.98	(0.64 to 1.52)	1.08	(0.87 to 1.35)	0.83	(0.65 to 1.07)	1.02	(0.89 to 1.18)	0.92	(0.82 to 1.03)
1–2 per week	1.06	(0.62 to 1.81)	1.15	(0.88 to 1.51)	0.73	(0.53 to 0.99)	1.01	(0.85 to 1.20)	0.94	(0.81 to 1.08)
3 times or more per week	1.03	(0.55 to 1.93)	0.95	(0.68 to 1.31)	0.97	(0.68 to 1.38)	1.11	(0.90 to 1.35)	0.82	(0.70 to 0.98)
p-val trend		0.82		0.98		0.47		0.45		0.07

Model adjusted for maternal educational level, parental financial situation, parental countries of birth, maternal and paternal age, and child's sex, age, and number of siblings

Children in the highest category of mobile phone use at age 5 were more likely to have less favourable sleep duration, more night wakenings and more parasomnias. Children in the highest mobile phone use category were also more likely to show higher bedtime resistance, one of our three selected negative control outcomes (Table 5; minimally-adjusted results are presented in S3 Table). Cordless phone use was not associated with any of the sleep scores. Mobile phone ownership at age 7 (n = 108, 5%) was associated with less problematic sleep onset delay (S2 Table). In adjusted models, total sleep duration in hours per day was not related to exposure to RF-EMF (data not shown).

**Table 5:** Fully-adjusted association between RF-EMF exposure sources and those sleeping problems *a priori* hypothesized NOT to be potentially related to RF-EMF exposure.

	Sleeping problems							
	Bedtime resistance		Sleep anxiety		Sleep disordered breathing		Total score	
	OR	(95% CI)	IRR	(95% CI)	IRR	(95% CI)	IRR	(95% CI)
**Environmental RF-EMF exposure from mobile phone base stations at home**								
<50th perc.	1.00		1.00		1.00		1.00	
50-90th perc.	1.06	(0.89 to 1.26)	1.08	(0.93 to 1.27)	0.94	(0.76 to 1.16)	1.03	(0.96 to 1.11)
>90th perc.	0.96	(0.73 to 1.28)	0.96	(0.74 to 1.24)	0.64	(0.44 to 0.94)	0.93	(0.83 to 1.04)
p-val trend		0.88		0.79		0.05		0.60
**RF-EMF indoor sources (Cordless phone/Wi-Fi)**								
None	0.69	(0.44 to 1.09)	0.59	(0.39 to 0.92)	1.09	(0.65 to 1.81)	0.96	(0.81 to 1.15)
WiFi yes, cordless phone no	0.90	(0.73 to 1.12)	0.90	(0.74 to 1.09)	1.42	(1.11 to 1.82)	0.92	(0.84 to 1.00)
WiFi no, cordless phone yes	0.94	(0.67 to 1.32)	0.70	(0.51 to 0.98)	1.27	(0.85 to 1.90)	0.90	(0.79 to 1.04)
Both	1.00		1.00		1.00		1.00	
**Mobile phone use at age 5**								
No use	1.00		1.00		1.00		1.00	
less than once per week	1.26	(1.06 to 1.50)	1.19	(1.02 to 1.39)	1.05	(0.85 to 1.30)	1.11	(1.03 to 1.19)
1–2 per week	0.98	(0.69 to 1.39)	0.83	(0.59 to 1.16)	0.98	(0.64 to 1.51)	1.08	(0.94 to 1.24)
3 times or more per week	2.08	(1.41 to 3.06)	1.36	(0.94 to 1.97)	1.15	(0.70 to 1.89)	1.30	(1.10 to 1.54)
p-val trend		<0.001		0.14		0.63		<0.001
**Cordless phone use at age 5**								
No use	1.00		1.00		1.00		1.00	
less than once per week	0.95	(0.74 to 1.22)	0.96	(0.77 to 1.20)	1.04	(0.76 to 1.41)	0.95	(0.86 to 1.05)
1–2 per week	1.21	(0.89 to 1.64)	0.93	(0.71 to 1.23)	1.21	(0.84 to 1.75)	0.96	(0.85 to 1.08)
3 times or more per week	0.96	(0.67 to 1.38)	0.93	(0.67 to 1.28)	1.25	(0.82 to 1.90)	0.90	(0.78 to 1.04)
p-val trend		0.48		0.60		0.16		0.23

Model adjusted for maternal educational level, parental financial situation, parental countries of birth, maternal and paternal age, and child's sex, age, and number of siblings.

Comparing residential environmental RF-EMF exposure to combined residential and school exposure yielded high overlap between levels of exposure (88% agreement of being categorised as low, medium or high exposure). Accordingly, analysing our *a priori* selected sleep subscales on the subgroup of children where we also had information on classroom exposure produced similar results (data not shown).

Sensitivity analysis on the additional sleep difficulties subscales also provided associations: Children of parents that had neither a WiFi, nor a cordless phone at home were reported to have less sleep anxiety (Table 5). Increased mobile phone usage at age 5 was also associated with higher bedtime resistance and the total sleep score (Table 5).

Finally, adjusting the analysis on mobile phone usage at age 5 on the subscales sleep onset delay, sleep duration and parasomnias for whether the child lived with both of his parents or not, usual amount of TV watching, playing computer games and playing outside in hours at age 5 during week days, asthma as well as the total behaviour problems score or asthma did not materially affect the risk estimates (data not shown).

## Discussion

The present study assessed the effect of RF-EMF exposure at age 5 from several sources on sleep problems in children at 7 years of age. Of five evaluated sleep scores, only higher exposure levels from mobile phone base stations (>90th percentile) was associated with less favourable sleep duration and with fewer parasomnias. Exposure to indoor sources was not associated with sleep scores, except absence of the indoor sources, which was associated with one of the control sleep scales, sleep anxiety. Regarding mobile phone and cordless phone use, less favourable sleep onset delay, sleep duration and parasomnias were increased with higher levels of mobile phone use. Mobile phone usage was also associated with one of our control sleep subscales, bedtime resistance. Higher level of cordless phone use was not associated to any of our sleep scales.

Strength of our study was that we could evaluate in a comprehensive way if exposure to RF-EMF was related to reported sleep quality in children, and we did so in a relatively large sample of children. We did not perform measurements, but were able to model environmental exposure to RF-EMF from base stations at home (and school) and asked several exposure-relevant questions, including the presence or absence of indoor sources such as cordless phones or wireless internet connections. We validated the exposure model beforehand, using both outdoor as well as indoor measurements, and found our model to perform reasonably well in predicting the relative ranking of exposure to the downlink component of mobile phone base stations [24, 25]. Also, the presence or absence of indoor sources like cordless phones and wireless internet connections has been previously shown to be a predictor of total (environmental) RF-EMF exposure in addition to owning an own mobile phone [32]. All of these predictors were also assessed in our analysis and we therefore feel that we were able to capture the most relevant sources of exposure to RF-EMF.

We predicted environmental RF-EMF exposure based on the residential (and school) address at age five but assessed sleeping problems at age seven. Although exposure patterns to RF-EMF may change with the introduction of new technologies, RF-EMF exposure from base stations has been previously shown to remain relatively stable at least over the time period of up to 10 months [24]. Obviously, residential exposure to base stations changes when persons move place of residence. A total of 198 (8%) of the children in our analysis moved between the two questionnaire surveys. However, using the modelled exposure for the last address, or omitting these children from our analysis, did not materially affect our estimates (data not shown).

We observed that children with higher environmental exposure to RF-EMF from mobile phone base stations had a somewhat increased risk of less favourable sleep duration. Of note, and although not statistically significant, children of parents with neither a cordless phones nor a wireless internet connection had a similarly increased score of sleep duration (i.e. worse sleep duration), although the absence of these sources should translate into lower total exposure to RF-EMF [32, 33]. Given the amount of analyses performed, potential alternative explanations for the observed association therefore also include chance, or else residual confounding.

A limitation of our study is the use of self-reported mobile phone use and sleeping problems assessed in the same questionnaire, which corresponds more to a cross-sectional design. Our observed association of the child’s mobile phone use with several of the sleep scores might therefore have also been produced by reporting bias: If parents were aware of any potential association, then we cannot exclude that parents of badly sleeping children were over-reporting mobile phone use compared to parents of children who slept better. However, it would be unclear why such reporting bias would affect only mobile phone use, but not cordless phone use. Another limitation of our study is the assessment of the child’s mobile phone use by parental reports. Previous studies have validated self-reported use of mobile phones with measurements, for example by comparing self-report with measured use as assessed with software-modified phones or operator data. Most of these studies have resulted in moderate to good correlation between reported and true use, with a tendency to underestimate number of calls and overestimate duration of calls [34–36]. However, to the best of our knowledge, parental reports of mobile phone use back in time have not been previously validated.

We observed that children for whom a higher usage level of mobile phones was reported were more likely to have worse sleep scores on three of our five *a priori* selected sleep subscales. Cordless phones have a much lower output power as compared to mobile phones, but do not have power control, that up- (UMTS) or down- (GSM) regulate output power of mobile phones until optimal reception is achieved. While it is therefore difficult to tell whether it is calling with mobile or cordless phones that adds most to RF-EMF exposure, both would be expected to contribute to peak head exposure [37]. Accordingly, at least similar patterns in the risk estimates across mobile and cordless phone use would be expected, also because both types of phones were reported to be used in similar amounts. Given that we observed no effect on sleep scores from the use of cordless phones but only with mobile phones, this suggests another underlying mechanism than exposure to RF-EMF. Alternative mechanisms that have been suggested to underlie the association between electronic media use and impaired sleep in children are the displacement of sleep by media use, physiological arousal when using media in the evenings or bright (blue) light from screens suppressing melatonin [22]. In contrast to cordless phones, mobile phones may also be used for other activities, such as gaming or texting. Seven year-old children are in the process of learning to read and write and therefore it is unlikely that impaired sleep was caused by being awakened from text messages, as has been observed in adolescents [38, 39]. Unfortunately, we do not know if mobile phones were also used for games, or the contents and time such games were played, which has been shown to be of relevance for quality of child sleep [40]. Mobile phone use was also related to bedtime resistance, indicating that this exposure may also be related to other factors, such as parenting skills.

In conclusion, the children exposed to higher levels of base station exposure had worse sleep scores of sleep duration but improved sleep scores of parasomnias, but we did not observe a similar effect in children exposed to RF-EMF from indoor sources. Also, we observed more sleeping problems in children that were reported to be higher users of mobile phones. This association was not present in children reported to be higher users of cordless phones. Given the differences of results across the evaluated RF-EMF exposure sources, our study does not provide support for the hypothesis that it is the exposure to environmental RF-EMF that is detrimental to sleep quality in 7-year old children, but rather other factors that are related to mobile phone usage as such. Our observation is in line with previous publications that have reported effects of electronic media use on children’s sleep.

## Supporting Information

S1 Table Minimally-adjusted association between RF-EMF exposure sources and those sleeping problems *a priori* hypothesized to be potentially related to RF-EMF exposure. Models adjusted for child's sex and age.
(PDF)

S2 Table Minimally as well as fully-adjusted association between mobile phone ownership at age 7 and sleeping problems. Minimally adjusted: model adjusted for child's sex and age. Fully adjusted: model adjusted for maternal educational level, parental financial situation, parental countries of birth, maternal and paternal age, and child's sex, age, and number of siblings.
(PDF)

S3 Table Minimally-adjusted association between RF-EMF exposure sources and those sleeping problems *a priori* NOT hypothesized to be potentially related to RF-EMF exposure. Models adjusted for child's sex and age.
(PDF)
