# Supplementary material for: Environmental Radiofrequency Electromagnetic Fields Exposure at Home, Mobile and Cordless Phone Use, and Sleep Problems in 7-Year-Old Children
Source: PLoS One. 2015 Oct 28;10(10):e0139869. doi: 10.1371/journal.pone.0139869 (PMC4625083; doi:10.1371/journal.pone.0139869)
Supplement: S1 Table — Models adjusted for child's sex and age. (PDF) [file pone.0139869.s001.pdf]

S1 Table: Minimally-adjusted association between RF-EMF exposure sources and those sleeping problems *a priori* hypothesized to be potentially related to RF-EMF exposure

	Sleeping problems									
	Sleep onset delay		Sleep duration		Night wakenings		Parasomnias		Daytime sleepiness	
	OR	(95% CI)	IRR	(95% CI)	IRR	(95% CI)	IRR	(95% CI)	IRR	(95% CI)
Environmental RF-EMF exposure from mobile phone base stations at home										
<50th perc.	0.00		1.00		1.00		1.00		1.00	
50-90th perc.	1.04	(0.78 to 1.37)	1.17	(1.01 to 1.34)	1.12	(0.95 to 1.32)	1.08	(0.99 to 1.19)	1.04	(0.96 to 1.12)
>90th perc.	0.97	(0.62 to 1.54)	1.31	(1.06 to 1.63)	0.92	(0.70 to 1.20)	0.88	(0.76 to 1.03)	0.99	(0.87 to 1.12)
p-val trend		0.98		0.005		0.82		0.78		0.71
RF-EMF indoor sources (Cordless phone/Wi-Fi)										
None	1.19	(0.66 to 2.15)	1.53	(1.16 to 2.03)	1.43	(1.02 to 2.01)	1.13	(0.93 to 1.39)	1.28	(1.08 to 1.51)
WiFi yes, cordless phone no	1.14	(0.82 to 1.58)	0.95	(0.80 to 1.13)	1.25	(1.03 to 1.51)	1.04	(0.93 to 1.16)	1.02	(0.93 to 1.12)
WiFi no, cordless phone yes	1.19	(0.72 to 1.98)	1.09	(0.84 to 1.41)	0.92	(0.67 to 1.25)	0.96	(0.81 to 1.14)	1.00	(0.86 to 1.15)
Both	1.00		1.00		1.00		1.00		1.00	
Mobile phone use at age 5										
No use	1.00		1.00		1.00		1.00		1.00	
less than once per week	1.02	(0.76 to 1.35)	1.17	(1.01 to 1.34)	1.14	(0.97 to 1.34)	1.09	(1.00 to 1.20)	1.04	(0.97 to 1.13)
1-2 per week	1.53	(0.93 to 2.53)	1.35	(1.03 to 1.77)	0.94	(0.67 to 1.32)	0.93	(0.77 to 1.12)	1.13	(0.97 to 1.32)
3 times or more per week	1.67	(0.96 to 2.92)	1.48	(1.09 to 2.00)	1.90	(1.36 to 2.65)	1.34	(1.10 to 1.64)	1.17	(0.98 to 1.39)
p-val trend		0.05		0.001		0.002		0.02		0.03
Cordless phone use at age 5										
No use	1.00		1.00		1.00		1.00		1.00	
less than once per week	0.96	(0.65 to 1.42)	1.05	(0.85 to 1.29)	0.81	(0.64 to 1.02)	1.05	(0.92 to 1.20)	0.93	(0.83 to 1.04)
1-2 per week	0.93	(0.57 to 1.52)	1.09	(0.85 to 1.39)	0.74	(0.56 to 1.00)	1.03	(0.87 to 1.21)	0.93	(0.81 to 1.06)
3 times or more per week	0.85	(0.48 to 1.52)	1.01	(0.75 to 1.35)	1.18	(0.85 to 1.63)	1.15	(0.95 to 1.39)	0.94	(0.80 to 1.10)
p-val trend		0.58		0.83		0.57		0.25		0.44

Models adjusted for child's sex and age.
