# Supplementary material for: Environmental Radiofrequency Electromagnetic Fields Exposure at Home, Mobile and Cordless Phone Use, and Sleep Problems in 7-Year-Old Children
Source: PLoS One. 2015 Oct 28;10(10):e0139869. doi: 10.1371/journal.pone.0139869 (PMC4625083; doi:10.1371/journal.pone.0139869)
Supplement: S2 Table — Minimally adjusted: model adjusted for child's sex and age. Fully adjusted: model adjusted for maternal educational level, parental financial situation, parental countries of birth, maternal and paternal age, and child's sex, age, and number of siblings. (PDF) [file pone.0139869.s002.pdf]

S2 Table Minimally as well as fully-adjusted association between mobile phone ownership at age 7 and sleeping problems

**Mobile phone ownership at age 7**		**Sleep onset delay**		**Sleep duration**		**Night wakenings**		**Parasomnias**		**Daytime sleepiness**	
		**OR**	**(95% CI)**	**IRR**	**(95% CI)**	**IRR**	**(95% CI)**	**IRR**	**(95% CI)**	**IRR**	**(95% CI)**
no	minimally adjusted	1		1		1		1		1	
yes		0.46	(0.21 to 1.00)	0.78	(0.56 to 1.09)	1.19	(0.83 to 1.69)	1.17	(0.96 to 1.42)	1.12	(0.95 to 1.33)
p-val			0.03		0.15		0.34		0.13		0.17
no	fully adjusted	1		1		1		1		1	
yes		0.29	(0.09 to 0.96)	0.72	(0.49 to 1.07)	0.84	(0.54 to 1.29)	1.03	(0.82 to 1.30)	1.01	(0.83 to 1.23)
p-val			0.02		0.10		0.43		0.77		0.93

Minimally adjusted: model adjusted for child's sex and age. Fully adjusted: model adjusted for maternal educational level, parental financial situation, parental countries of birth, maternal and paternal age, and child's sex, age, and number of siblings.
